# The Effect of Light Rail Transit on Physical Activity: Design and Methods of the Travel-Related Activity in Neighborhoods Study

**DOI:** 10.3389/fpubh.2016.00103

**Published:** 2016-06-09

**Authors:** Casey P. Durand, Abiodun O. Oluyomi, Kelley Pettee Gabriel, Deborah Salvo, Ipek N. Sener, Deanna M. Hoelscher, Gregory Knell, Xiaohui Tang, Anna K. Porter, Michael C. Robertson, Harold W. Kohl

**Affiliations:** ^1^Department of Health Promotion and Behavioral Science, University of Texas Health Science Center at Houston School of Public Health, Houston, TX, USA; ^2^Michael & Susan Dell Center for Healthy Living, University of Texas Health Science Center at Houston School of Public Health, Austin, TX, USA; ^3^Department of Epidemiology, Human Genetics and Environmental Sciences, University of Texas Health Science Center at Houston School of Public Health, Austin, TX, USA; ^4^The University of Texas Health Science Center at Houston School of Public Health, Austin, TX, USA; ^5^Center for Nutrition and Health Research, National Institute of Public Health of Mexico, Cuernavaca, Mexico; ^6^Texas A&M Transportation Institute, Austin, TX, USA; ^7^Department of Health Promotion and Behavioral Science, University of Texas Health Science Center at Houston School of Public Health, Austin, TX, USA; ^8^University of Texas at Austin, Austin, TX, USA

**Keywords:** transportation, physical activity, active travel, light rail, built environment, transit

## Abstract

**Background:**

Use of mass transit has been proposed as a way to incorporate regular physical activity into daily life because transit use typically requires additional travel to access and depart the stop or station. If this additional travel is active, a small but potentially important amount of physical activity can be achieved daily. Although prior research has shown that transit use is associated with physical activity, important questions remain unanswered. Utilizing a major expansion of the Houston, TX, USA light-rail system as a natural experiment, the Houston Travel-Related Activity in Neighborhoods (TRAIN) Study was developed to address these unanswered questions.

**Purpose:**

The purpose of the TRAIN Study is to determine if the development of light-rail lines in Houston, TX, USA will prospectively affect both transit use and physical activity over 4 years. We also aim to understand how contextual effects (i.e., moderators or interaction effects), such as the neighborhood built environment and socioeconomic factors, affect the primary relations under study.

**Methods:**

The TRAIN Study is a longitudinal cohort design, in which participants are recruited at baseline from a 3-mile buffer around each of the three new lines and measured annually four times. Recruitment is accomplished via telephone contact, ads in newspapers and advertising circulars, and targeted community outreach. Data are collected via mail and include questionnaire-assessed factors, such as perceived neighborhood characteristics, attitudes about transportation, demographics, and reported physical activity; a travel diary; and accelerometry. Additionally, field-based neighborhood audits are conducted to capture micro-scale environmental features. To assess macro-scale environmental characteristics, we utilize GIS mapping and spatial analyses. Statistical analyses will be conducted using latent growth curve modeling and discrete choice models, with a focus on identifying moderating factors (i.e., statistical interaction effects). Selection bias will be controlled via propensity score analysis.

**Conclusion:**

The TRAIN study is a unique opportunity to study how a multi-billion dollar investment in mass transit can simultaneously affect transportation needs and physical activity behavior. This comprehensive evaluation will provide needed evidence for policy makers, and can inform health impact assessments of future transportation projects around the world.

## Background and Rationale

Lack of physical activity continues to be a significant public health concern. In the United States, only 4% of adults are achieving 30 min per day of physical activity, and major disparities exist across racial/ethnic groups and by socioeconomic status (1, 2). According to the 2008 Physical Activity Guidelines for Americans, regular physical activity is strongly associated with maintaining a healthy weight, and is also associated with a lower risk of cardiovascular disease, type 2 diabetes, and colon, breast, and endometrial cancers (3). Regular physical activity among cancer survivors has also been associated with improved quality of life (3). Due to its association with obesity and chronic disease, significant time and effort has been spent in an attempt to identify the underlying determinants of physical activity.

Scientific study of physical activity behavior has historically focused on psychosocial factors (e.g., self-efficacy, norms, attitudes, etc.) that enable or hinder health-promoting physical activity (4, 5). Although some behavioral interventions have found success in promoting physical activity, preventing sedentary lifestyles, or reducing weight, population levels of physical activity have remained stubbornly low. It is not surprising, then, that over the past 10–15 years there has been increasing awareness that factors outside the individual, including policy and the built environment (defined as all features of the external world constructed or modified by humans), may play a prominent role in physical activity behavior (6, 7).

Transportation infrastructure has been a part of the built environment for many years, but especially since personal ownership of an automobile in the US became commonplace. Over time, the bulk of transportation spending and policies at all levels of government shifted from favoring mass transit, such as bus or rail lines, to favoring cars and suburban, single family residential development (8). National data reflect this shift: public transit trips declined by 56% from 1945 to 2010 (9). Meanwhile, the number of personal vehicles increased by 181% from 1969 to 2001 (10). At the same time, land development patterns shifted to accommodate the increased prominence of personal cars. New development is frequently characterized by segregated land uses and accessibility only by car (11–13). The end result is that we have engineered out of our daily lifestyles the need to walk or bike to destinations, such as work, school, or shops.

Recognizing the simultaneous dependence on automobiles and population-wide decrease in physical activity, some have argued in recent years for a re-emergence of mass transit as an effective way to incorporate regular physical activity into people’s daily lives (14–16). The major reason for this is that transit use typically requires some interim travel from departure location to the transit stop and to the destination. This distance is typically on the order of 0.25–0.50 miles (17). For an average person, this distance equates to a comfortable walking time of 10–15 min (18, 19). For someone using transit to get to and from work or a store, this translates to four separate bouts of activity, for a total of about 40–60 min per round-trip. If walking at a moderate intensity, even irregular transit use would contribute a large proportion of the U.S. Department of Health and Human Services (DHHS) recommended 150 min per week of moderate-intensity physical activity (3).

Light rail transit (LRT) is a form of mass transit characterized by electric powered trains running fixed routes along dedicated track corridors, generally with traffic signal priority to increase efficiency (20, 21). Passengers board at dedicated stations, rather than from the sidewalk like a traditional bus stop. Because they are smaller than commuter-type trains, they have greater utility for implementation in dense urban areas (21). LRT use in the United States has increased by 280% from 1990 to 2010 as measured by passenger miles, a greater increase than any other form of transit (22).

Light rail transit has particular promise for increasing physical activity. First, LRT stops are typically further apart than stops for bus lines, requiring greater amounts of walking or biking to reach them (16, 17). Second, LRT has the potential to encourage construction of mixed-use transit-oriented development to cluster around its stops, particularly in urban areas (20, 21). Not only do these developments increase the attractiveness of using LRT, it also may have an additional benefit of encouraging destination-based walking to housing or retail options not previously available to local residents, even those who do not use LRT. Third, compared to bus service, LRT is able to more rapidly connect multiple population and employment clusters within a single city (21). Decentralized development of this nature is characteristic of many of the fastest growing cities in the United States. This increases LRT’s attractiveness to car-driving individuals who may be reluctant to spend an extended period of time on a bus to reach one of these clusters. Fourth, this increased connectedness and accessibility to the rest of the city may be especially attractive to lower income individuals who lack cars. Greater accessibility to destinations has repeatedly been linked to increased amounts of transportation-related physical activity (23, 24).

Despite the promise of a link between LRT and physical activity, there is a lack of data from controlled, long-term studies in this area. To address this research gap, we developed the Houston Travel-Related Activity in Neighborhoods (TRAIN) Study. The purpose of the TRAIN Study is to determine if the development of light-rail lines in Houston, TX, USA will prospectively affect both transit use and various domains of physical activity in racially/ethnically diverse and low-income communities over 4 years. We also aim to understand how contextual effects (i.e., moderators or interaction effects), such as the neighborhood built environment and socioeconomic factors, affect the primary relations under study.

## Methods

### Conceptual Model

**Figure 1:**
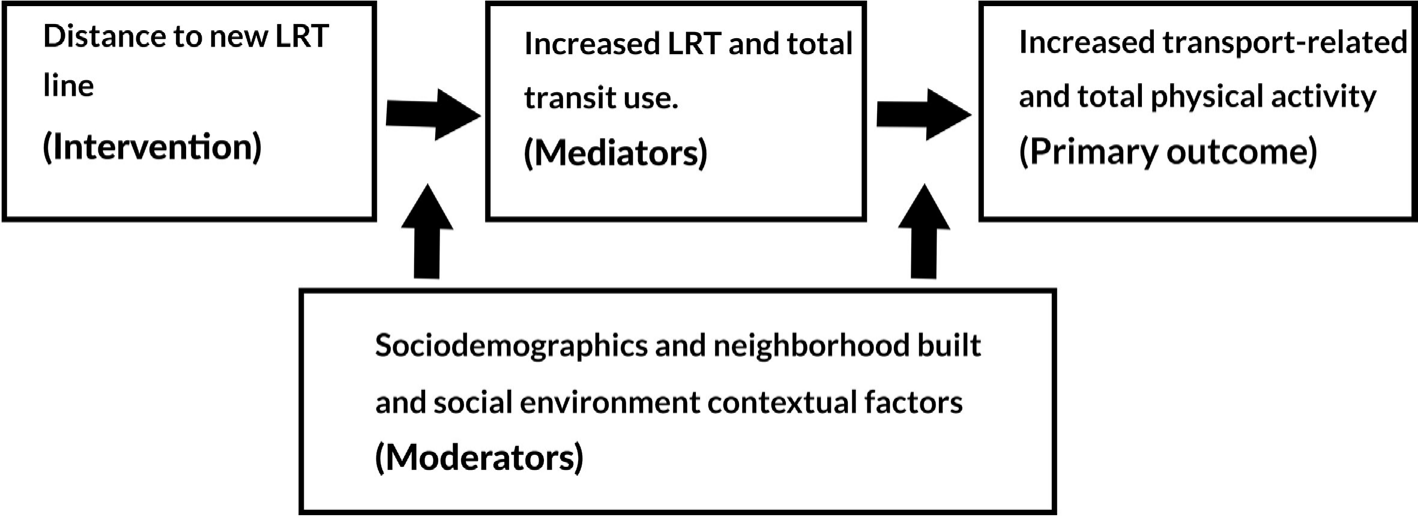
**Conceptual model of the association between light rail transit, transportation, and physical activity**.

Overall, the TRAIN study is grounded in a social-ecological model (SEM) of behavior. According to SEM, behavior is a function of intra-individual, inter-individual, social, community, environmental, and policy factors (25). As such, our study approach and measurement methods are designed to capture relevant factors at each level, as well as the relations among those factors. A conceptual model of how LRT may affect physical activity is shown in Figure 1. The overall aim of the TRAIN Study is to understand how access to LRT affects transportation behavior, and how that behavior in turn affects physical activity, represented in Figure 1 by paths “1” and “2.” However, we are interested in going beyond these questions to further understand the context in which any changes occur. Of prime interest are interaction or moderation effects, denoted in Figure 1 by the paths labeled “3.” These effects describe the conditions under which changes do or do not occur. For example, it may be that access to LRT is more strongly linked to transit use in neighborhoods in which there is a greater perceived sense of safety from crime. Or it may be possible that men who use transit are more likely to walk to the station than women, who may be more likely to be dropped off by a friend or family member.

### Study Setting

**Figure 2:**
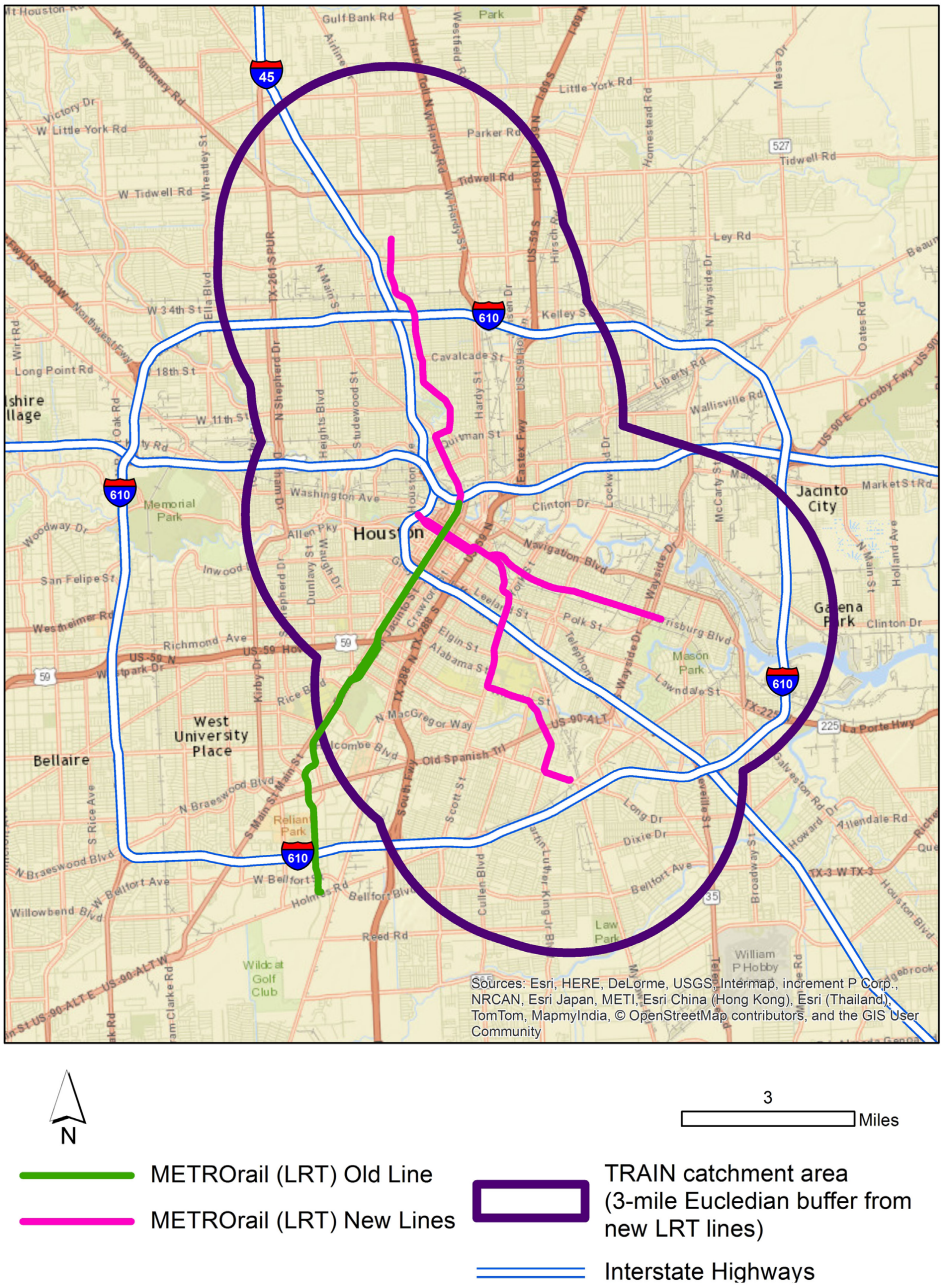
**Map of the TRAIN study catchment area and METROrail system**.

The study takes place in Houston, TX, USA, the fourth largest city in the United States. As a general matter, Houston lacks what could be described as a culture of public transportation, and instead has invested (along with state and federal governments) heavily in highway and toll way projects. Beginning in 2004, however, the Metropolitan Transit Authority of Harris County (METRO), the agency overseeing public transit in Harris County, opened its first light-rail line. The system was subsequently expanded, with new lines opening in December 2013 and May 2015, resulting in 15 miles of new LRT lines and 24 new stations (see Figure 2). Unlike the original line, which ran primarily through office and medical districts, the new lines run through primarily residential and light commercial areas. Importantly, the population served by the new light-rail lines is primarily minority (Black/African American and Hispanic), and low income. This is the same population that is more likely to be physically inactive, obese, or overweight, and suffer from chronic health conditions (26, 27).

The target area for the study is defined by an airline buffer (i.e., a direct or “crow-fly” distance) extending 3 miles out from either side of the new rail lines (Figure 2). This buffer was chosen to maximize our pool of eligible participants and to provide a diversity of distances from home to the LRT lines, our primary predictor of LRT use. There is currently an inexact understanding of the distances individuals are willing to travel, and in particular, walk, in order to reach public transit. Generally, transit planners have based transit-stop catchment zones on the assumption that individuals will not walk more than about a quarter-mile to a stop (28). However, more recent research demonstrates that individuals may be willing to walk much further, perhaps even several miles, in order to reach a transit stop (28). Given that, we felt a larger buffer would in effect capture a range of probabilities in terms of willingness to walk to the light-rail stops.

### Participants and Recruitment

Sample size calculations indicated a need for ~ 750 participants at baseline. This calculation was based on the following assumptions: desired power of 90%, alpha of 0.05, 9% participant attrition per year, four waves of data collection, and effect size equivalent to 0.3 SD units. Monte Carlo simulation methods were used to perform the sample size calculations in the context of the latent growth curve (LGC) models proposed for analysis (29).

Inclusion criteria were that an individual had to be at least 18 years old, reside within the defined study buffer area, and only one participant per household was allowed. No other inclusion/exclusion criteria were used (e.g., they did not have to be transit users, there were no upper age limits, or any limits on disability status). Study participants were recruited using different methods and channels. Initially, a market research company was employed to match telephone numbers (landline and cellular) and email addresses with residential addresses within the study buffer. Using this information, potential participants were then contacted, the study was explained to them, and they were asked if they would like to participate. Upon acceptance, they were classified as “provisional participants,” and data collection procedures were initiated. However, after 6 months of recruitment using this method alone, enrollment results were deemed less than satisfactory. We then decided to supplement this recruitment method with targeted community outreach. This included the following strategies: attending community events to promote the study (e.g., civic association meetings, farmer’s markets, National Night Out, parent–teacher meetings, church services, and food fairs), distributing flyers to apartment complexes, announcements in local advertisement circulars, referrals from existing participants, and door-to-door recruitment.

Prospective participants were given the option of two forms of participation: the “basic” group completed the self-report survey and a 2-day travel diary, and were compensated with a $25 gift card for their time. The “enhanced” group completed the self-report survey, a 7-day travel diary, and wore an accelerometer during the same 7-day period as the travel diary; they were compensated with a $50 gift card for their time. Participants were notified that they would be asked to participate once a year for 4 years, but could opt out of participation at any point in time. Informed consent was obtained from all participants. The institutional review boards of the University of Texas Health Science Center Houston and Texas A&M University reviewed and approved the study protocol.

### Participant-Derived Measures

#### Self-Report Survey

A paper questionnaire consisting of approximately 80 questions was used to assess characteristics, behaviors, and psychosocial constructs previously linked to travel patterns, physical activity, and/or obesity, all of which are guided by the SEM (6). These included attitudes and perceptions about different forms of transportation, reasons for use or non-use of transit, reasons for choosing their current home, importance of neighborhood features if they were to move, usual destinations visited, expectations regarding changes to their neighborhood resulting from the LRT expansion, self-rated health, body weight, medical conditions, disability status, and sociodemographic factors.

#### Travel Diaries

Travel diaries were used to gather information about participant’s travel patterns. Daily travel was assessed with the use of a standard travel diary (from 03:00 a.m. on the travel day through 03:00 a.m. the following day). The “basic” group participants opted to fill a 2-day travel diary (one weekday and one weekend day), while the remaining “enhanced” participants were asked to fill a full week (7-day) travel dairy. For each trip on their assigned travel diary days, participants were asked to record details about their trips during the survey days, such as the purpose of trip (work, shopping, recreation, etc.), mode of transportation (car, LRT, bus, walk, bike, etc.), how long trip took in minutes, time of day, and day of week of trip. Participants were asked to record all trips, regardless of trip duration or length.

#### Self-Report Physical Activity

The paper questionnaire included self-report measures of physical activity and sedentary behavior, including the Modifiable Activity Questionnaire (S-MAQ) and Multi-Context Sitting Time Questionnaire (MSTQ). The S-MAQ is a self-administered survey that assesses leisure-time and transport-related physical activities over the past 7 days. The structure of the leisure-time physical activity component of the S-MAQ is similar to the interviewer-administered versions of the MAQ (i.e., past year and past week) and includes information on 38 activity types common among this population sub-group (30–32). Leisure-time physical activity levels were calculated as the product of the duration and frequency of each activity (in hours per week), weighted by an estimate of the metabolic equivalent (MET) of that activity and summed across all activities performed (33). The transportation component included recall prompts specific to walking, bicycling, and other modes (e.g., skateboarding) for active transport and participants were allowed to report up to four one-way trips per day of observation. Similar to the leisure-time component, transport-related physical activity was calculated as the product of the duration and frequency of each active transport type (in hours per week), weighted by the MET of that activity and summed across all modes of active transport (33). Summary estimates for both the leisure-time and transportation-related components are expressed as MET hours per week (MET h week^−1^). The MET values range from 2.5 (Bowling) to 9.0 (Jumping Rope, Martial Arts, Racquetball/Handball/Squash). The past-week MAQ has been shown to be a reliable and valid measure of physical activity (31, 32).

The MSTQ is a self-administered survey that was designed to capture the amount of time spent (hours and minutes) sleeping and engaging in five contexts of sitting during a typical work and non-work day (34). The contexts of sitting used as cues for recall were selected to capture the majority of daily sitting within the leisure-time, occupation, and transportation domains. Participants were asked to report sitting time for a typical work and non-work day. Prior to scoring, time values were converted from hours and minutes to total minutes. Then, sitting time across contexts was summed to obtain the summary estimates of total sitting time during a typical work and non-work day. The MSTQ has been previously been shown to be reliable and valid in similar populations (34).

#### Accelerometers

Participants were mailed an accelerometer data collection package that included a tri-axial accelerometer (ActiGraph GT3X+), secured to an adjustable elastic belt. The accelerometer data collection package also included (1) an introductory letter, (2) detailed written instructions, (3) a list of frequently asked questions with responses, (4) instructions on how to access an instructional YouTube video that detailed proper accelerometer wear (https://goo.gl/Iv59rm), (5) an Activity Monitor Tracking Log, (6) a Participant Checklist to assist the return of data collection materials, and (7) a pre-paid, addressed padded envelope for participants to return data collection materials to study staff.

Participants were asked to wear the accelerometer on their right hip, secured by an adjustable elastic belt, during all waking hours for seven consecutive days. For each day of data collection, participants were also asked to record the times in the Activity Monitor Tracking Log corresponding to when she/he (1) put on the accelerometer in the morning, (2) took the accelerometer off at night, and (3) took the accelerometer off for 15 min or longer and put it back on during the day. Based on pilot work, it was determined that the expected delivery time from Austin, TX, USA (field center site) to Houston, TX, USA via the U.S. Postal Service (USPS) was 2 days. Therefore, the accelerometers were initialized to begin data collection at midnight, 2 days after they were mailed and to continue collecting data until the devices were downloaded by study staff. Raw accelerometer data were sampled at 40 Hz and data were reintegrated prior to further processing. An accelerometer monitor retrieval protocol was triggered when the completed accelerometer package was not returned to study staff when expected. More specifically, reminder postcards were mailed and phone calls were made to participants at 1 week, and again 2 weeks, after the package was expected to be delivered to the participant. If the completed accelerometer package was not returned to study staff within 4 weeks of expected returned date (14 days post sent-date), study staff would telephone the participant weekly, for a period of 3 weeks, to request the data collection package and troubleshoot, as needed.

Accelerometer data were screened for periods of wear (i.e., wear time) using established wear-time algorithms (35). Briefly, non-wear time was defined as 90 consecutive minutes of zero counts, with an allowance of 2 min of non-zero counts provided there were 30-min consecutive zero count windows up- and down-stream (35). All accelerometer estimates were derived using the vertical axis data. Total accelerometer counts per day (ct d^−1^) were calculated using summed daily counts detected over wear periods. Time spent per day (minutes per day) in different intensity levels were estimated using count threshold values proposed by Freedson et al. (36). These are sedentary [0–99 counts per minute (ct⋅min^−1^)], light- (100–1951 ct min^−1^), moderate- (1952–5724 ct min^−1^), and vigorous- (≥5725 ct min^−1^) intensity. Summary estimates of time spent per day in moderate–vigorous physical activity (MVPA) were also computed using thresholds of ≥1952 ct min^−1^. The first MVPA estimate included every minute above threshold (accumulated MVPA); whereas the second estimate (MVPA in bouts) only included accumulated time spent in bouts of at least 10-min duration (1). Weekly summary estimates were computed by averaging daily estimates across total number of days worn for all participants with ≥4 of 7 days with ≥10 h per day of valid wear time.

### Environmental Measures

#### Perceived Environment

Participants were asked to self-report on a number of environmental measures. For example, we sought to characterize perceptions of many aspects of the built environment itself, including access to destinations, esthetics, traffic safety, and sidewalk/bicycle routes (37). This measure also included questions about the social environment, such as interactions with neighbors, and whether there are people out and about in the neighborhood. Perceptions about crime-related safety and victimization are assessed by asking about how often a participant worried about becoming a victim of certain crimes. We also asked specific questions about perceptions of physical and social disorder, including litter/trash in streets, poorly maintained property, drinking in public, and people fighting/arguing (38).

#### Distance to the New Light-Rail System (Main Exposure of Interest)

To determine the effect of the new light-rail lines on transit use and physical activity, the exposure of interest was defined as the “distance to the new light-rail system (from residential location).” Therefore, this study uses a continuous exposure variable (distance from home to the new light-rail lines), the hypothesis being that those residing closer to the new light-rail lines will increase their transit-related physical activity relative to those residing at further distances from the LRT. As previously described, all TRAIN participants reside (at baseline) within a 3-mile airline buffer from the new light-rail lines, which provides a wide range of distances between each participant’s residential address and their nearest light-rail lines, thus yielding high variability in the exposure variable to better address the research questions of interest. To give some perspective, at average walking speed, it would take an adult 1 h to walk 3 miles. Therefore, the study sample ranges from participants residing within less than a 5-min walk to the new light rails (higher exposure), up to an hour walk away (lower exposure). To operationalize the exposure, several indicator variables will also be created using participant’s residential addresses and geoprocessing techniques with geographic information systems (GIS) to determine the distance from each participant’s residential location to the new transit lines. For instance, we will calculate crow-fly distances from the participant’s home to the nearest LRT line (point-to-line airline distance), crow-fly distances from the participant’s home to the nearest LRT station (point-to-point airline distance), as well as network-distances, i.e., nearest route using the street network, versus simple airline distance, both to the nearest line (point-to-line network distance) and to the nearest LRT station (point-to-point network distance).

#### Other GIS-Derived Neighborhood and Travel Route Characteristics

A series of built environment variables are being constructed using GIS, which will allow us to address the question of whether the relations between transit use and physical activity among participants with access to transit (distance to the LRT) are moderated by built environment features. The concept of a “neighborhood” is complex (39, 40), and the optimal spatial definition for a neighborhood in terms of size and shape has not been defined (40, 41). As such, there are several commonly used spatial unit definitions for a “neighborhood” in the physical activity and built environment literature (42–46). We will construct GIS variables using both an administrative boundary approach (census blocks, block groups, and tracts), and an individualized, participant-centered approach (street-network buffers). Participant home addresses as well as reported frequent destinations (obtained via study survey and travel diaries) will be geocoded and spatially linked to administrative neighborhood units. The geocoded addresses will also be used to construct network buffers of varying radii (500, 1000, and 1500 m) to generate individual, participant-centered spatial neighborhood units. The main advantage of the administrative boundary approach is that most publically available GIS data are aggregated using these administrative units. On the other hand, the individualized street-network approach places participants in the center of the “neighborhood” and is able to capture the built environment features that participants can actually access through the road network (46). We will create GIS variables to measure characteristics of the built environment that have been reported to be associated with physical activity and behaviors (43, 44, 47). These include, but are not limited to: residential density, street connectivity, land-use mix, park density, tree canopy coverage, public transit-stop density, sidewalk length and coverage, and bicycle lane length and coverage. The spatial and tabular data necessary for operationalizing the built environment GIS-derived variables have been obtained primarily through public sources, including the US Census, the City of Houston GIS portal, the City of Houston Department of Transportation, among others. All GIS variables are being geoprocessed using ArcGIS 10.3 software (48).

#### Environmental Audit

The *Active Neighborhood Checklist* (Analytic) was used to collect data on micro-scale built environment features for the TRAIN study (49). This measure is completed by trained study staff. The Checklist has been validated for use in conducting environmental audits, and already successfully used in previous studies (50–53). It was developed as an observational tool to be used for the assessment of key street-level features of the neighborhood environment that are thought to be related to physical activity behavior. The Checklist covers major built environment themes, including land use, public transit stops, street characteristics, quality of the environment for a pedestrian, and places to walk and bicycle. For the TRAIN study, auditors were trained on the use of the Checklist using a customized training manual that was developed by the TRAIN study team. The audit exercise was conducted over the course of 8 weeks, between May 15, 2014 and July 15, 2014. The method for selecting the location for the audit exercise was systematically arranged. Importantly, our goal was to assess the built environment features that are within the close proximity of the LRT stations. The rationale for this approach was twofold: first, we are interested in determining, at baseline, whether street segments that are close to the stations have built environment features that are supportive of active commuting; second, we are interested in determining whether the built environment features surrounding these stations will change significantly over the course of the TRAIN study (5 years).

**Figure 3:**
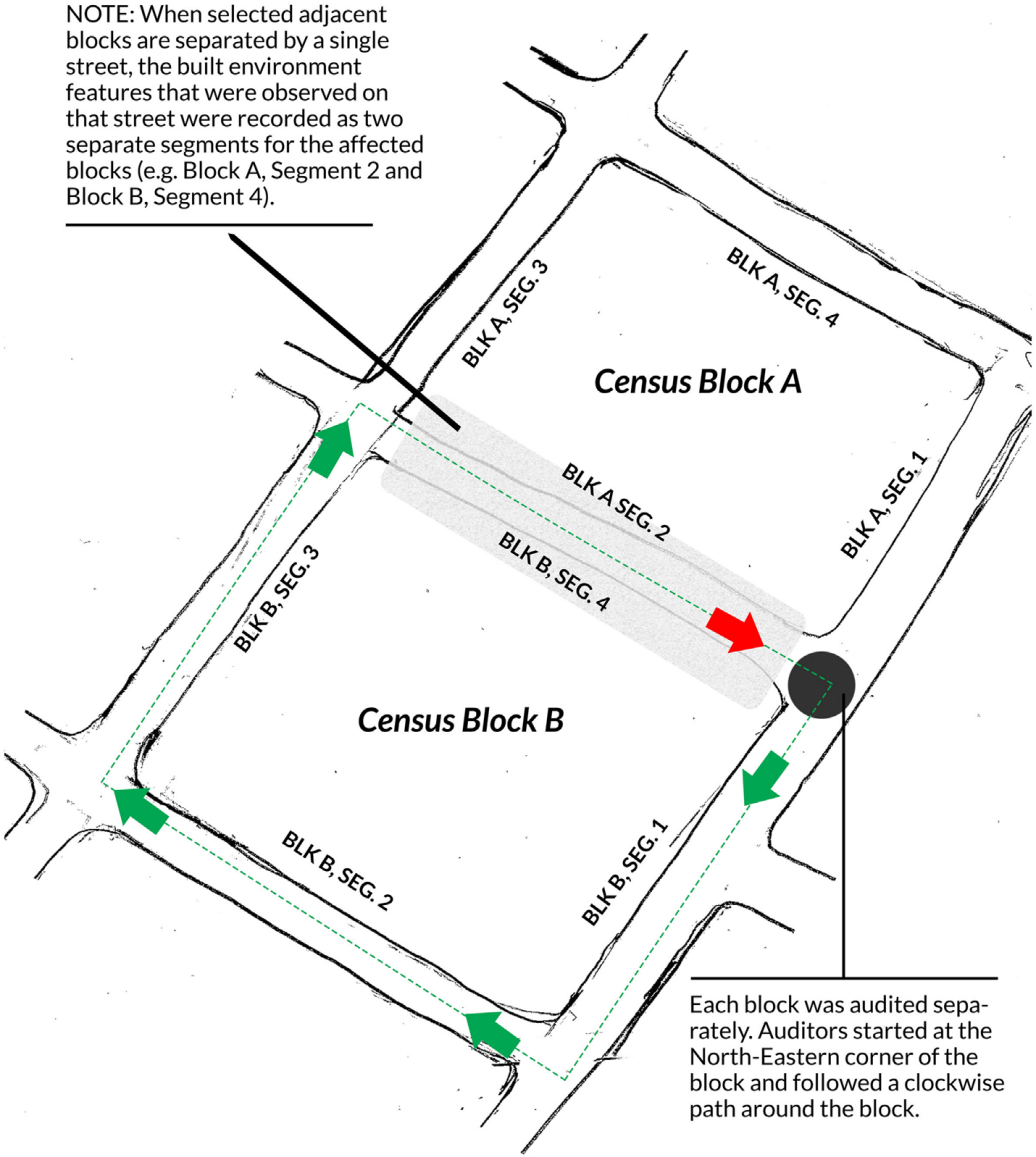
**Map demonstrating typical environmental audit procedures**.

The protocol that we used to select the segments to audit was based on using proximity analysis in ArcGIS. Essentially, we used the location of the station as the focal point for creating a 0.5-mile circular buffer around the station. We then selected between 8 and 15 census blocks that are contained inside the buffer. These were used for our environmental audit. A typical census block has four segments; which are the streets that bound the census block. For the purpose of the audit, the segment for a given block is the block-facing features on any given street that also act as a boundary for the block. Figure 3 shows a typical set of segments surrounding two adjacent blocks. As depicted in the figure, the features that were recorded for Block A Segment 2 and Block B Segment 4 were sourced from the same street. Any street that borders two blocks of interest will be recorded as two separate segments; once for each block that it borders. To illustrate, features on both sides of “A Street” are recorded as segment “S2” for block 1 and segment “S4” for block 2. In all, a total of 599 segments were audited from 22 LRT stations.

The majority of audit exercise was completed on a hand-held Android-based tablet. The paper version of the checklist instrument was transcribed into Qualtrics, an electronic platform for serving and completing surveys online or offline (54). Auditors completed the surveys on the tablets as they would if using paper and pen. On a typical audit day, five teams of at least two people participated in the audit fieldwork.

### Data Collection Procedures

Data collection for the TRAIN study is mail-based, and takes place in four waves: baseline data collection, 1-year follow-up, 2-year follow-up, and 3-year follow-up. Upon recruitment and eligibility verification (baseline), prospective participants were notified via phone or email that a study packet would be sent to their mailing address. For subsequent waves of data collection, participants are contacted by phone at 12, 24, and 36 months post baseline to notify them that the study packet for the given wave (2, 3, and 4, respectively) will be delivered and to verify their address. The TRAIN study survey is designed for self-administration, is available in English or Spanish versions, and takes approximately 90 min to complete.

To maximize protocol compliance and study material (survey, travel diary, accelerometer, and log-form) returns, a follow-up phone-based protocol is implemented. Two weeks after mailing the packet, participants receive a phone-call by trained study personnel, prompting them to complete the survey and return their study materials. Up to six subsequent calls are made (maximum one call per week), at which point a letter is sent to the participant’s mailing address offering a final opportunity to return their study materials and continue to participate in the study.

Detailed, item-by-item protocols were developed to screen the survey, travel diary, and accelerometer-log data prior to data entry. Upon reception of print materials at the study coordinating center, they are screened by trained study personnel. Data entry is done by trained personnel using REDCap (Research Electronic Data Capture) software (55). Upon completion of data entry at each wave, a 5–10% random sample was selected and verified against the printed instrument to ensure data entry accuracy.

### Analysis Plan

To assess the hypothesized relationship between proximity to LRT and LRT use over time (path 1 in Figure 1), LGC modeling will be used (56). LGC is a highly flexible technique, and can model not only linear relationships but also curvilinear ones, such as quadratic (57). It can also be structured as a piecewise model, such that discrete periods of time can have markedly different slopes (58). It can further accommodate latent, or unobserved, factors, and a variety of fit statistics are available to determine whether the hypothesized model adequately fits the observed data (59). Individual times of observation are allowed to vary, and there is no requirement that measurement times be equally spaced. Also, our analyses will use full information maximum likelihood (FIML) estimation, so that each participant is not required to have complete data; all available data can be used. Similar methods will be used to understand the relation between transit use and physical activity (path 2 in Figure 1). To understand the full mediation model represented by paths 1 and 2 combined, we will further construct a parallel process growth curve model that allows us to formally test the hypothesis of a causal chain linking transit access, transportation behavior, and physical activity. If necessary, we can also accommodate clustering of participants within the LGC models, such as clustering around LRT stations, or within neighborhoods, census tracts, zip codes, etc.

We can further extend this model by examining whether any of the demographic, social, or built environment factors interact with LRT exposure to differentially affect transit use, or interact with transit use to differentially affect physical activity (path 3 in Figure 1). That is, we can determine whether any moderating effects are present. Depending on whether the hypothesized moderating variable is discrete or continuous, this can be accomplished with either a multiple group LGC model or by adding an interaction term in the LGC model (57).

In addition to the longitudinal data methods that may be familiar to many public health researchers, we will also incorporate state-of-the-practice methodologies used in the transportation research field. The statistical models developed here will particularly be based on discrete choice modeling frameworks; discrete choice models are used to analyze and predict a decision maker’s choice of one alternative from a finite set of mutually exclusive and exhaustive alternatives (60).

One consistent concern noted in evaluations of environmental characteristics on physical activity is selection bias. In general, this would occur if more physically active individuals self-select into neighborhoods that facilitate their active lifestyles. This affects the ability to discern whether the environment is actually causing behavior. Specific to transit studies, and in the context of current study, active individuals may choose residences based on their desire to accrue physical activity by walking or biking to and from transit stops. To some degree, our study area limits selection bias; duration of residence and age of housing stock in the target areas is lengthy, and there is not a significant amount of new construction occurring at this point. Nevertheless, without a randomized design (which is not feasible here), we cannot completely rule selection bias out. Therefore, we will additionally incorporate propensity scores into our models to statistically control for selection bias (61, 62). Propensity scores are a means to ensure a balance between groups on important confounders that may be the source of selection bias. In fact, propensity scores have been shown to produce a balance between groups on confounders almost as good as would result from a truly randomized design (62). A propensity score is defined as the probability an individual receives a treatment (here, proximity to one of the LRT corridors) conditional on a set of observed covariates. It is developed through a logistic regression model in which the LRT status (in corridor or control area) is the dependent variable, and variables hypothesized to contribute to selection bias are the predictors (61). This model results in the actual propensity score, which is one numerical value for each individual that is included as a covariate, for example, in the case of the LGC model by regressing the latent growth factors on it.

## Conclusion

Research on the manner in which the built environment may affect health behaviors is a new and exciting field. We have designed the Houston TRAIN Study to provide significant new insight into the specific question of how our transportation system affects physical activity over time. With a multi-disciplinary research team, a rich, longitudinal, multi-level dataset, and a target population of much interest, we believe our study is well-positioned to provide a detailed analysis that can assist in future health impact assessments, multimodal transportation planning, policy making, and behavioral interventions to promote utilitarian travel as an important source of physical activity.

## Author Contributions

CD led the conceptualization and design of the project, and drafted the manuscript. AO, KG, DH, IS, and HK contributed to the conceptualization and design of the project, and assisted in drafting the manuscript. DS, GK, XT, AP, and MR contributed to project implementation, and assisted in drafting the manuscript.

## Conflict of Interest Statement

The authors declare that the research was conducted in the absence of any commercial or financial relationships that could be construed as a potential conflict of interest.
